# Wheat F-Box Protein Gene *TaFBA1* Is Involved in Plant Tolerance to Heat Stress

**DOI:** 10.3389/fpls.2018.00521

**Published:** 2018-04-24

**Authors:** Qinxue Li, Wenqiang Wang, Wenlong Wang, Guangqiang Zhang, Yang Liu, Yong Wang, Wei Wang

**Affiliations:** State Key Laboratory of Crop Biology, Shandong Key Laboratory of Crop Biology, College of Life Sciences, Shandong Agricultural University, Tai’an, China

**Keywords:** F-box protein, heat stress, wheat, gene expression, transgenic tobacco

## Abstract

Adverse environmental conditions, including high temperature, often affect the growth and production of crops worldwide. F-box protein, a core component of the Skp1-Cullin-F-box (SCF) E3 ligase complex, plays an important role in abiotic stress responses. A previously cloned gene from wheat, *TaFBA1*, encodes a homologous F-box protein. A Yeast two-Hybrid (Y2H) assay showed that TaFBA1 interacted with other SCF proteins. We found that the expression of *TaFBA1* could be induced by heat stress (45°C). Overexpression of *TaFBA1* enhanced heat stress tolerance in transgenic tobacco, because growth inhibition was reduced and photosynthesis increased as compared with those in the wild type (WT) plants. Furthermore, the accumulation of H_2_O_2_, O_2_^-^, and carbonyl protein decreased and cell damage was alleviated in transgenic plants under heat stress, which resulted in less oxidative damage. However, the transgenic plants contained more enzymatic antioxidants after heat stress, which might be related to the regulation of some antioxidant gene expressions. The qRT-PCR analysis showed that the overexpression of *TaFBA1* upregulated the expression of genes involved in reactive oxygen species (ROS) scavenging, proline biosynthesis, and abiotic stress responses. We identified the interaction of TaFBA1 with *Triticum aestivum* stress responsive protein 1 (TaASRP1) by Y2H assay and bimolecular fluorescence complementation (BiFC) assay. The results suggested that TaFBA1 may improve enzymatic antioxidant levels and regulate gene expression by interacting with other proteins, such as TaASRP1, which leads to the enhanced heat stress tolerance seen in the transgenic plants.

## Introduction

Many different environmental cues affect plant growth and development, of which extreme temperature and water deficit are two major factors (Fang et al., 2015). It has been reported that the increased temperature caused by global warming will have adverse effects on crop yields throughout the 21st century (Sato et al., 2016). To cope with these stress conditions, plants need to adopt a series of efficient strategies, which ultimately lead to morphological, physiological, and biochemical changes (Fang et al., 2015). The regulation of gene expression is an important strategy used by plants for adapting to heat stress. Some genes, including those encoding transcription factors and functional proteins, are involved in heat stress tolerance of plants. For instance, *Arabidopsis thaliana* has a positive regulator of dehydration-responsive element binding protein 2A (DREB2A), the transcriptional regulator DNA polymerase II subunit B3-1 (DPB3-1), and nuclear factor Y subunit C10 (NF-YC10), which play vital roles in abiotic stress tolerance. In addition, the overexpression of *DPB3-1* enhances heat stress tolerance without growth retardation. Furthermore, *DPB3-1*-overexpressing rice showed enhanced heat stress tolerance (Sato et al., 2014, 2016). *SNAC3* is a stress-responsive NAC gene and its overexpression in rice resulted in enhanced tolerance to high temperature, drought, and oxidative stress, whereas suppression of SNAC3 by RNAi led to contrasting phenotype when exposed to these stresses (Fang et al., 2015).

In all eukaryotes, the ubiquitin/26S proteasome system is very important in selectively degrading intracellular proteins (Moon et al., 2004; Smalle and Vierstra, 2004; Ho et al., 2008). In this system, the polymeric ubiquitin chains recognized and modified intracellular proteins via an ATP-dependent reaction cascade. Then these intracellular proteins are degraded by the 26S proteasome. Three enzymes participated in the ubiquitination of a target protein. These are the E1 ubiquitin-activating enzymes, the E2 ubiquitin-conjugating enzymes, and the E3 ubiquitin ligases (Hershko and Ciechanover, 1998; Yan et al., 2013; Zhou et al., 2015). SKP1/CUL1/F-box protein (SCF) E3 ligase is the best characterized class of E3 ubiquitin ligases, among which F-box protein is a key subunit protein that is involved in responses to abiotic stresses (Moon et al., 2004). Hua and Vierstra (2011) reported that the C-terminal WD40 motif or the leucine-rich repeat region of F-box protein binds targeted substrates to provide reaction specificity.

Some papers have reported E3 ligases function in heat response. For example, the expression of the plant E3 ligase BnTR1 could significantly increase the thermotolerance of *Escherichia coli* (*E. coli*) and BnTR1 expression induced the accumulation of heat shock proteins. Niu et al. (2016) reported that their findings indicated the expression of BnTR1 confers thermoprotective effects on *E. coli* cells. SIZ1 is a well-characterized Small Ubiquitin-like Modifier (SUMO) E3 ligase, Zhang et al. (2017) reported that SUMO conjugations were clearly induced by high temperature. Overexpression of SIZ1 SUMO E3 ligase (SlSIZ1) could enhance the tolerance to heat stress in tomato and the level of heat-shock protein (HSP) 70 at high temperatures was increased in transgenic plants. The rice (*Oryza sativa*) *HEAT TOLERANCE AT SEEDLING STAGE* gene, *OsHTAS*, is a RING Finger Ubiquitin E3 Ligase and plays a positive role in heat tolerance in rice. Liu et al. (2016) suggested *OsHTAS* functions in leaf blade to enhance heat tolerance through modulation of hydrogen peroxide-induced stomatal closure. Also, *Oryza sativa* Heat and Cold Induced 1 (*OsHCI1*) is a RING E3 ligase gene which is highly up-regulated by heat and cold stress. The ectopic expression of YFP fused OsHCI1 in *Arabidopsis* showed a heat-tolerant phenotype and increased survival rate under heat stress (Lim et al., 2013). Moreover, it has been reported that the F-box protein transport inhibitor response 1 (TIR1) auxin co-receptor accumulated rapidly after being induced by increased temperature, and this response was dependent on the molecular chaperone HSP90 (Wang R.H. et al., 2015). *AtCHIP* is a U-Box-Containing E3 Ubiquitin Ligase gene in *Arabidopsis*, Yan et al. (2003) stated the transcript of *AtCHIP* was up-regulated by high temperatures. The overexpression of *AtCHIP* in *Arabidopsis* rendered plants more sensitive to high-temperature treatments. All those suggested the important role of different type E3 ligases in the regulation of plant heat tolerance.

Wheat is a universal and important food crop, and improving its resistance to stresses has become an important research theme around the world. Previously, we obtained an F-box gene *TaFBA1* from wheat (*Triticum aestivum* L.). The expression of this gene in wheat was induced by different stresses, including NaCl, drought and ABA treatment in wheat (Zhou et al., 2014). We transformed this gene into tobacco to improve our understanding of the role played by *TaFBA1* in plant stress tolerance, and to gain insight into the mechanism underlying its control. We found that the transgenic tobacco lines overexpressioning *TaFBA1* displayed an ameliorative performance when exposed to drought (Zhou et al., 2014) and salt stresses (Zhao et al., 2017), and the oxidative stress tolerance of the transgenic plants was increased (Zhou et al., 2015). In the present study, we analyzed how TaFBA1 affected heat tolerance. The possible molecular mechanisms underlying this phenomenon were studied by detecting the interaction of TaFBA1 with other proteins.

## Materials and Methods

### Plant Materials and Growth Conditions

Tobacco plant variety NC 89 (*Nicotiana tabacum* cv) was used as the wild type (WT). According to the methods described previously by Zhou et al. (2014), the transgenic tobacco plants were produced and identified. Three homozygous transgenic tobacco lines: (OE), OE-8, OE-15, and OE-18, were selected. The WT and OE lines were grown in vermiculite under greenhouse conditions with a photoperiod of 16-h light/8-h dark and at 25°C. The germinating wheat seeds were placed on a homemade gauze that could be installed on enamel trays filled with Hoagland solution. Then the seeds were grown under a 16-h-light/8-h-dark cycle at 25°C and 70% relative humidity (light intensity 300 μmol m^-2^ s^-1^).

### Stress Treatments

After the wheat seedlings had been grown hydroponically in Hoagland solution for a week, they were subjected to 45°C heat treatment. The second leaf was harvested at 0, 0.5, 1, 2, 3, 6, 9, 12, and 24 h after 45°C treatment and then the mRNA expression levels of *TaFBA1* and abiotic stress response protein 1 (TaASRP1) were detected by real-time PCR.

Eight-week-old tobacco plants were used to analyze the expression profiles of stress-related genes. The *NtFBW2* and *NtASRP1* responses to heat stress were analyzed by exposing the tobacco plants to 45°C for 0, 0.5, 1, 2, 3, 6, 9, 12, and 24 h in the light.

Eight-week-old tobacco plants were also exposed to 45°C for 6 h in the light and then their plant physiological parameters were measured. After heat treatment at 45°C for 6 h, the adult tobacco plants were moved to normal condition (16-h-light/8-h-dark cycle at 25°C) to grow for 3 days, and then recovery plants were obtained.

Furthermore, 14-day-old tobacco plants were exposed to 45°C for 6 h to analyze reactive oxygen species (ROS) accumulation.

### Root Length Measurements

Wild type and T3 transgenic tobacco seeds were processed using 75% ethanol for 2 min and then disinfected with 3% sodium hypochlorite (NaClO) for 10 min. All the sterilized seeds were rinsed in sterilized deionized water at least five times and moved to MS plates to grow for a week. For heat stress, the plates containing the plants were exposed to 45°C for 24 h. After that, they were subjected to normal growth conditions for 5 days, while the control seedlings were moved to normal condition (16-h-light/8-h-dark cycle at 25°C) all the time. Finally, their root lengths were measured. We randomly selected 20 seedlings from each line for fresh weight and root length analysis and the experiment was performed using three biological replicates, which produced similar results.

### Chlorophyll Content, Malondialdehyde Content, Electrolyte Leakage, and Photosynthesis-Related Parameter Assays

The chlorophyll content was measured using a UV spectrophotometric method as described by Yang et al. (2009). The malondialdehyde (MDA) content was measured according to Zhou et al. (2014) and electrolyte leakage was detected according to Lutts et al. (1996).

The photosynthetic gas exchange parameters of the tobacco leaves were measured using the procedure followed by Wang B.S. et al. (2015). The maximum photochemical efficiency of PSII (Fv/Fm) was measured according to Tian et al. (2013).

### Proline and Soluble Sugar Content Determination

The proline content was determined according to Ryu et al. (2014) and the soluble sugar content was determined according to Zong et al. (2009).

### Measurement of Antioxidative Enzyme Activities and O_2_^-^ and H_2_O_2_ Levels

The peroxidase (POD), catalase (CAT), superoxide dismutase (SOD), and ascorbate peroxidase (APX) activities were measured as described in a previous study by Wang et al. (2012).

The H_2_O_2_ accumulation was measured using 3,3′-diaminobenzidine (DAB) (Giacomelli et al., 2007). Qualitative testing of O_2_^-^ was undertaken using nitroblue tetrazolium (NBT) following the method from Rao and Davis (1999). The H_2_O_2_ concentration and O_2_^-^ production rate were measured according to Hui et al. (2012).

### Trypan Blue Staining Analysis Assay

The analysis of the trypan blue staining was carried out according to Koch and Slusarenko (1990).

### Immunological Analyses of HSP70 Abundance and Protein Carbonylation Assay

Eight-week-old transgenic tobacco and WT plants were subjected to 45°C heat treatment for 6 h. Then they were used in the immunological analyses of HSP70 abundance and protein carbonylation. Total protein was extracted from the tobacco leaves and its concentration was determined by a dye-binding assay (Bradford, 1976). Sodium dodecyl sulfate-polyacrylamide gel electrophoresis (SDS–PAGE) on 10% and 14% gradient gels were adopt to separate the proteins, and then the separated proteins were transferred to a PVDF membrane (Millipore, Saint-Quentin, France). After that, protein carbonylation was detected using the anti-dinitrophenyl (DNP) antibody (Sigma, St. Louis, MO, United States) and HSP70 proteins were detected with the HSP70 antibody (Sigma). The quantitative proteins analysis was performed on a Tanon GIS system (Tanon, Shanghai, China). The measurement of protein carbonylation levels was carried out according to Tian et al. (2014).

### Protein Interaction Assay

The yeast two-hybrid (Y2H) assays were performed as described previously (Lee et al., 2007). The complementary DNA (cDNA) fragments of Cullin were subcloned into the pGBKT7 or pGADT7 vectors. The cDNA fragments of Skp1 and TaFBA1 were subcloned into the pGBKT7 vector, respectively. And then they were co-transformed into the yeast strain Y2H Gold using the lithium acetate-mediated transformation method (Ito et al., 1983). Transformant candidates were selected on SD-Leu-Trp, SD-Leu-Trp-His, and SD-adenine-His-Leu-Trp media in order to evaluate the interaction between bait and prey proteins.

The BD Matchmaker library construction and screening kit (Clontech, Dalian, China) was used for the Y2H assays. The coding region of *TaFBA1* was inserted into the GAL4 DNA-binding domain vector pGBKT7 to generate a bait vector. The prey cDNA library was from wheat. The positive pGADT7 clones were selected on synthetic dextrose/-Leu/-Trp/-His/-Ade/5-Bromo-4-chloro-3-indolyl β-D-galactopyranoside plates (Wang et al., 2009; Chen et al., 2013) and then they were further identified by sequence. All protocols were carried out according to the manufacturer’s instructions.

For the bimolecular fluorescence complementation (BiFC) assay, TaFBA1 and TaASRP1 were respectively fused to the C terminus and the N terminus of YFP. Both constructs were coexpressed transiently in *Arabidopsis* leaf protoplasts via PEG transformation. The fluorescence signal was detected by confocal microscopy (Leica). The whole protocols were carried out referring to the methods in Tang et al. (2016).

### Quantitative RT-PCR Assay

qRT-PCR was carried out in a 25 ml reaction volume with Super Real Premix Plus (TIANGEN, Beijing, China). Quantitative analysis was performed using the CFX96 Touch^TM^ Real-Time PCR Detection System (Bio-Rad, United States). This method normalizes the expression of a specific gene versus a control reference with the formula 2^-ΔΔC_T_^. In this study, the mRNA levels for one stably expressed genes *NtActin*, were evaluated as control genes for qRT-PCR analyses. The information from all of the genes in the qRT-PCR experiments are listed in Supplementary Table S1.

### Statistical Analysis

All experiments and determinations were conducted at least in triplicate. The data processing system procedures (DPS, Zhejiang University, China) were used to perform statistical analyses. Statistical significance was evaluated using a *t*-test at the 0.05 or 0.01 probability levels.

## Results

### Confirmation That Wheat F-Box Protein TaFBA1 Is Part of the SCF Complex

**FIGURE 1:**
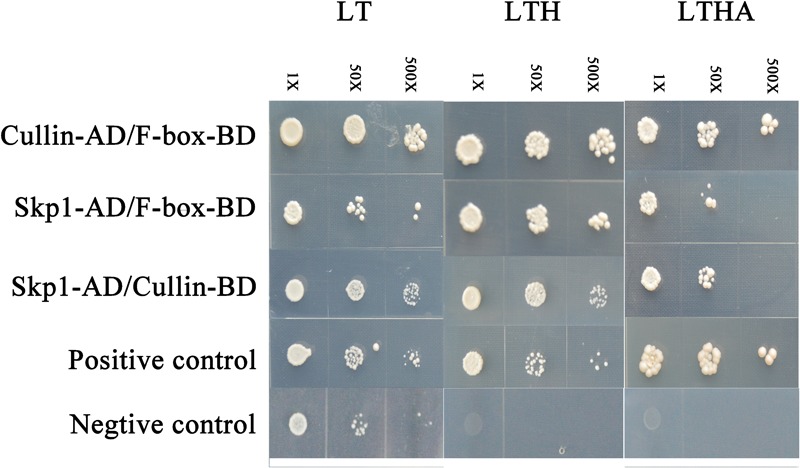
Identification of the interaction between TaFBA1 and other subunits in the Skp1-Cullin-F-box (SCF) complex by yeast two-hybrid (Y2H) analysis. The interactions between pGADT7-324 and pGBKT7-332, pGADT7-322, and pGBKT7-332 proteins were used as positive and negative controls. LT, LTH, and LTHA represent the selective media SD-Leu-Trp, SD-Leu-Trp-His, and SD-Leu-Trp-His-Ade, respectively.

We tested the interaction among Skp1 (GenBank: AY316293.1), Cullin (GenBank: EMS49792.1) and TaFBA1 to confirm that TaFBA1 (GenBank: JN038382.1) is the key subunit of the SCF complex. Y2H assay revealed that the three proteins could interact with each other (**Figure 1**). This suggested that the F-box protein coded by *TaFBA1* was a subunit of the SCF complex.

### *TaFBA1* Expression Pattern Responses to Heat Stress

**FIGURE 2:**
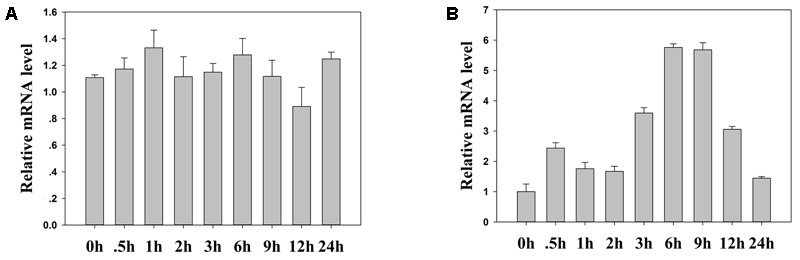
*TaFBA1* mRNA expression responses to heat stress in wheat. The qRT-PCR analysis of *TaFBA1* responses after 0, 0.5, 1, 2, 3, 6, 9, 12, and 24 h of normal **(A)** and 45°C **(B)** heat treatment by wheat that had been grown in Hoagland solution for 1 week. The data represent the mean ± SE of three biological replicates.

We investigated TaFBA1 mRNA expression using qRT-PCR to test whether *TaFBA1* expression in wheat changes in response to heat stress treatments. The *TaFBA1* transcription levels were analyzed in plants that had been grown under normal growth condition and 45°C heat at different time points (0, 0.5, 1, 2, 3, 6, 9, 12, and 24 h). The results in **Figure 2** illustrated that *TaFBA1* expression was gradually induced by heat stress, and the highest expression level was observed after 6 h of heat treatment. This suggested that *TaFBA1* may be involved in a heat responsive pathway.

### Overexpression of *TaFBA1* Improved Heat Tolerance in Transgenic Tobacco Seedlings

**FIGURE 3:**
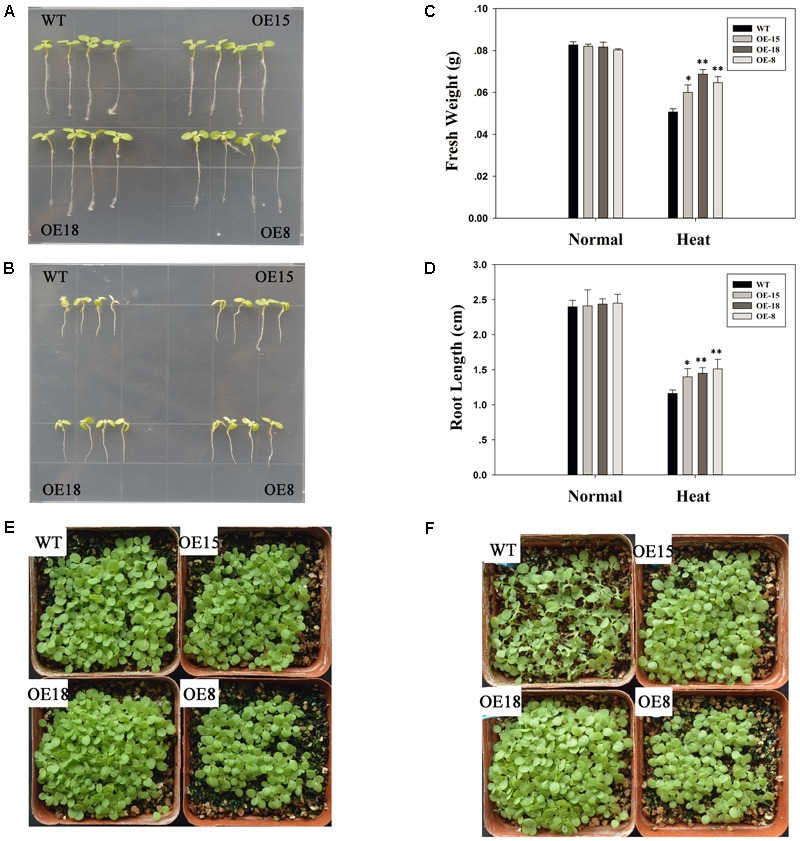
Overexpression of *TaFBA1* increased the heat tolerance of three transgenic tobacco lines at the seedling stage. **(A,B)** Growth status of single transgenic and wild type (WT) plants under normal growth conditions and after 45°C heat stress for 24 h, respectively. **(C)** Fresh weight of 20 seedlings. **(D)** Primary root lengths of the seedlings before and after heat treatment. **(E,F)** Phenotypes of 2-week old tobacco seedlings under normal conditions and after 45°C heat stress for 24 h. The data represent the mean ± SE of three biological replicates. ^∗^*P* < 0.05; ^∗∗^*P* < 0.01.

The transgenic tobacco lines: OE-8, OE-15, and OE-18, were used to confirm the roles of *TaFBA1* in heat stress tolerance responses. Eight-week-old WT and transgenic tobacco lines were kept at 45°C for 6 h and their responses to heat stress were examined. Both the WT and transgenic plants grew well before heat treatment and had reached similar growth stages (**Figures 3A,E**). However, after exposure to heat stress, significant leaf withering on the WT plants was observed (**Figure 3F**). The root lengths of the transgenic seedlings were also longer than those of the WT (**Figures 3B,D**). Furthermore, the fresh weights of the WT plants were consistently lower than those of the transgenic plants (**Figure 3C**).

### Overexpression of *TaFBA1* Improved the Growth and Photosynthetic Capacity of the Transgenic Plants Grown Under Heat Stress

**FIGURE 4:**
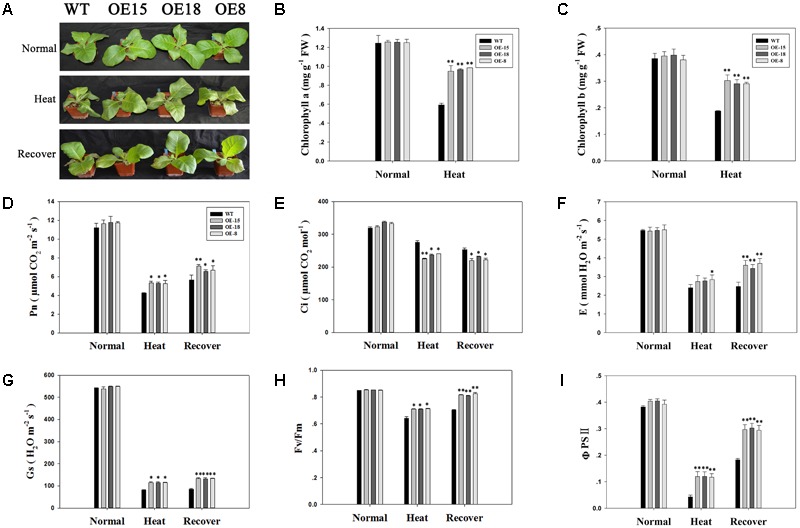
Overexpression of *TaFBA1* improved the growth and photosynthetic capacity of the transgenic plants grown under heat stress. **(A)** Phenotypes of 8-week-old tobacco plants after normal, 45°C heat stress for 6 h, and a 3 days heat stress recovery period. **(B,C)** Chlorophyll a and chlorophyll b contents, respectively. **(D)** Net photosynthetic rate (Pn), **(E)** intercellular CO_2_ concentration (Ci), **(F)** transpiration rate (E), **(G)** stomatal conductance (Gs), **(H)** maximum photochemical efficiency of PSII (Fv/Fm), and **(I)** actual PSII efficiency under irradiance (ΦPSII) of tobacco leaves from the WT plants and the transgenic lines under normal, heat treatment, and heat stress recovery conditions. The data represent the mean ± SE of three biological replicates. ^∗^*P* < 0.05; ^∗∗^*P* < 0.01.

Under normal conditions, transgenic and WT plants showed no significant phenotypic differences (**Figure 4A**). After heat treatment, the leaves of the plants showed different degrees of withering and green color loss. Leaves of transgenic plants were less withered than those of the WT plants (**Figure 4A**). Accordingly, the transgenic plants had higher average chlorophyll contents (**Figures 4B,C**).

The photosynthetic system is usually susceptible to damage induced by heat. Therefore, the effects of heat stress on photosynthetic gas exchange parameters were measured. The data in **Figure 4D** showed that there were no significant differences in the net photosynthesis rate (Pn) between transgenic and WT plants under normal conditions. After heat treatment, Pn decreased in all lines, but this decrease was lower in the transgenic lines than in the WT plants. After a 3 days recovery period, the Pn value increased slightly, but was still lower than that for the plants grown under normal conditions. However, the Pn of the transgenic plants was higher than that of the WT plants. The results for transpiration rate (E) (**Figure 4F**) and stomatal conductance (Gs) (**Figure 4G**) were consistent with those of Pn. Similar results were also recorded for Fv/Fm (**Figure 4H**) and ΦPSII (**Figure 4I**). However, the change in the intercellular CO_2_ concentration (Ci) showed a trend opposite to that of Pn (**Figure 4E**). The results shown in **Figure 4** demonstrated that the photosynthetic capacity of the transgenic plants was less sensitive to heat stress than that in the WT plants.

### Overexpression of *TaFBA1* Alleviated Reactive Oxygen Species (ROS) Accumulation and Heat-Induced Cell Damage

**FIGURE 5:**
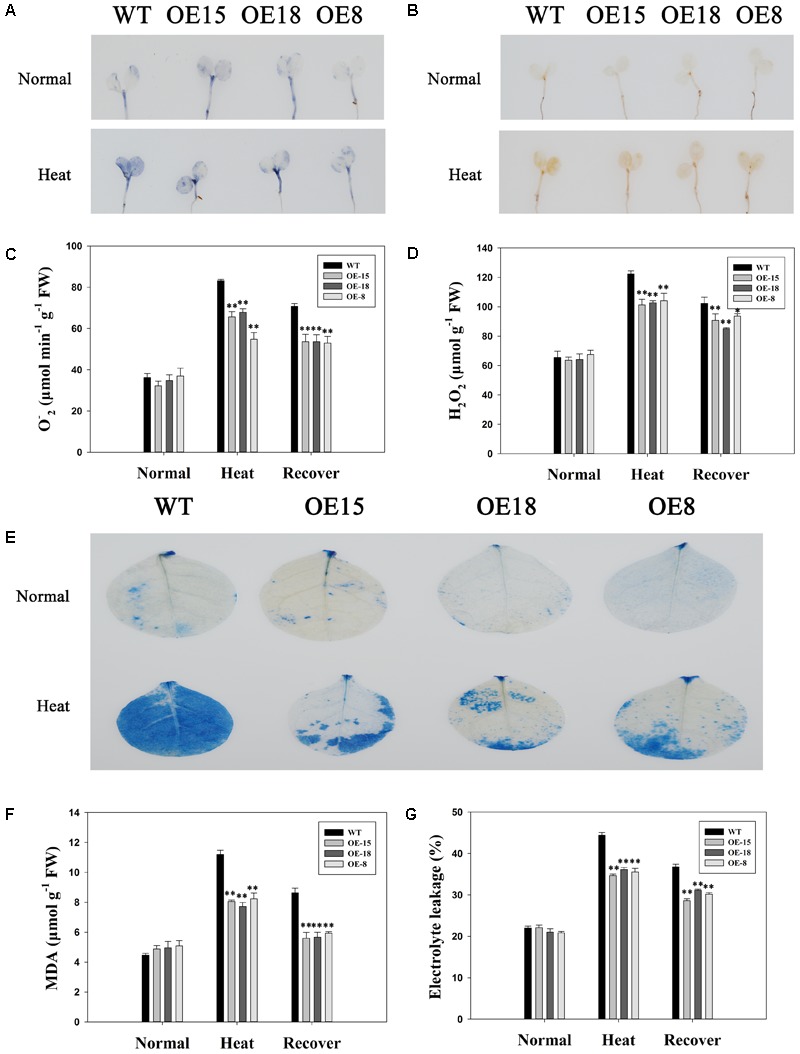
Reactive Oxygen Species (ROS) accumulation and cell damage in the WT plants and the transgenic lines. The H_2_O_2_ **(A)** and O_2_^-^ **(B)** accumulations were detected by histochemical staining using nitroblue tetrazolium (NBT) and 3,3′-diaminobenzidine (DAB), respectively. The top represents plants grown at 25°C and the bottom represents plants treated at 45°C for 24 h. Quantitative analysis of O_2_^-^ **(C)** and H_2_O_2_ **(D)**. **(E)** Trypan blue staining. The top represents plants grown at 25°C and the bottom represents plants treated at 45°C for 24 h. **(F)** malondialdehyde (MDA) content. **(G)** Electrolyte leakage. The data represent the mean ± SE of three biological replicates. ^∗^*P* < 0.05; ^∗∗^*P* < 0.01.

Reactive oxygen species are usually produced with aerobic respiration, and are usually considered to be toxic molecules that cause oxidative damage to DNA, proteins, and membrane lipids in plant cells (Fang et al., 2015). The intracellular levels of H_2_O_2_ and O_2_^-^ were analyzed by DAB and NBT staining, respectively (**Figures 5A,B**) to examine the ROS accumulation in both WT and transgenic plants under heat stress. Without heat treatments, both O_2_^-^ and H_2_O_2_ accumulation was low and there were no significant differences between the WT and transgenic plants. After exposure to heat stress, the ROS accumulation level increased in all the plants. However, less H_2_O_2_ and O_2_^-^ existed in the transgenic plants than in the WT plants. After a 3 days recovery period, ROS accumulation had slightly decreased in both the WT and transgenic lines, but was still higher than that under normal conditions (**Figures 5C,D**).

We used trypan blue staining to investigate the damage to cells. All plants showed similar levels of blue staining under normal growth conditions, but the WT lines showed darker blue staining than the transgenic lines under heat stress (**Figure 5E**). Ulteriorly, MDA accumulation and electrolyte leakage, which have been reported to be cell damage indicators (Wang G.D. et al., 2015) were assessed to verify that results. After heat treatment for 6 h, the transgenic plants had remarkably lower MDA contents and electrolyte leakage levels than the WT plants did, but there were no significant differences under normal conditions (**Figures 5F,G**), which suggested that the transgenic plants experienced less lipid peroxidation and were able to maintain a higher membrane stability than WT plants under heat stress.

### Overexpression of *TaFBA1* Reduced Protein Carbonylation Levels

**FIGURE 6:**
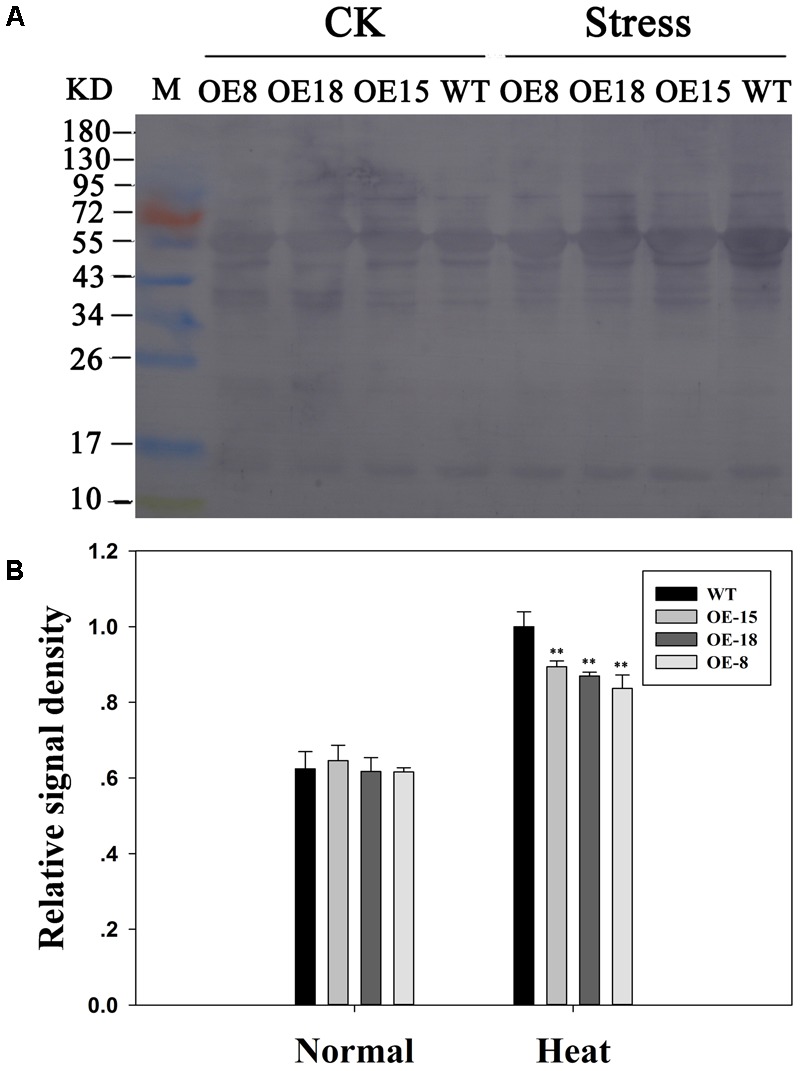
Effects of heat stress on protein carbonylation levels in the transgenic and WT plants. **(A)** Protein carbonylation. CK means normal growth condition at 25°C. Stress means plants after 45°C heat stress treatment for 6 h. **(B)** Relative signal density of the protein carbonylation levels. The data represent the mean ± SE of three biological replicates. ^∗^*P* < 0.05; ^∗∗^*P* < 0.01.

The role of TaFBA1 in coping with heat stress at the protein level was investigated by determining the protein carbonylation levels in the WT and transgenic plants using immunoblotting (**Figures 6A,B**). Both the transgenic and the WT plants performed increased carbonylated protein content after heat stress in **Figure 6B**. However, significantly less increase was found in the transgenic plants than in the WT plants.

### Overexpression of *TaFBA1* Increased Antioxidative Enzyme Levels Under Heat Stress

**FIGURE 7:**
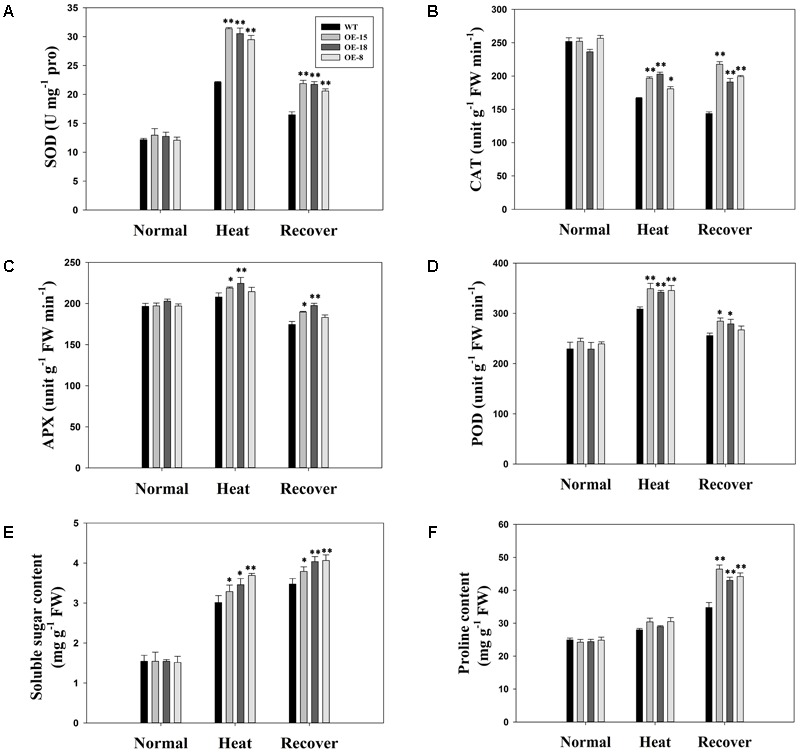
Antioxidative abilities and osmolyte accumulation under normal, heat treatment, and heat stress recovery conditions in the WT plants and the transgenic lines. **(A)** super oxide dismutase (SOD), **(B)** catalase (CAT), **(C)** ascorbate peroxidase (APX), and **(D)** peroxidase (POD) activities in 8-week-old tobacco plants. **(E)** Soluble sugar content and **(F)** proline content. The data represent the mean ± SE of three biological replicates. ^∗^*P* < 0.05; ^∗∗^*P* < 0.01.

We determined the enzyme activities of SOD, CAT, POD, and APX. There were no obvious differences in enzyme activity levels between the WT and transgenic plants before heat stress, but heat stress changed the activity levels of these enzymes in different ways (**Figures 7A–D**). For example, the SOD and POD activities were significantly increased by heat stress. After a 3 days recovery period, the activity levels of these two enzymes decreased slightly but they were still higher than those under normal conditions. In contrast, the CAT activity levels were reduced by heat stress. Additionally, APX activity was slightly, but not significantly, increased by heat stress. However, over all, the transgenic plants showed higher enzyme activities than the WT plants did after exposure to heat stress.

Proline and soluble sugars are typical osmolytes that can alleviate osmotic stress induced by heat stress. During heat stress, the concentration of both these osmolytes increased in all lines studied (**Figures 7E,F**). However, the *TaFBA1*-overexpressing lines had significantly higher soluble sugar contents than the WT plants (**Figure 7E**). No significant increases in proline content were recorded for the transgenic lines under heat stress (**Figure 7F**), however, during the recovery period, both the proline and soluble sugar contents remained high (**Figures 7E,F**).

### *TaFBA1* Overexpression Altered the Expression Levels of Some Stress-Responsive Genes

**FIGURE 8:**
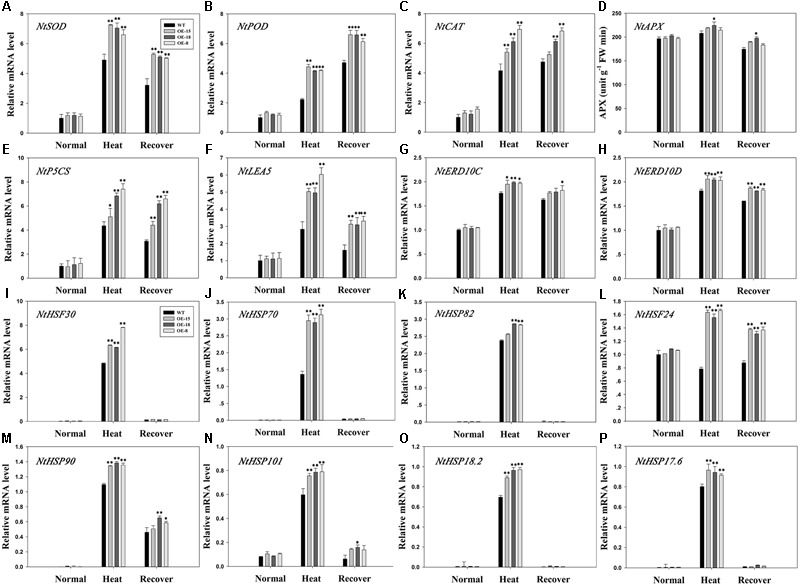
Relative expressions of antioxidant-related and stress-responsive genes in the WT and transgenic plants. Antioxidant-related genes: *NtSOD* **(A)**, *NtPOD* **(B)**, *NtCAT* **(C)**, and *NtAPX* **(D)** responses to heat stress. Stress-responsive genes: *NtP5CS* **(E)**, *NtLEA5* **(F)**, *NtERD10C* **(G)**, *NtERD10D* **(H)**, *NtHSF30* **(I)**, *NtHSP70* **(J)**, *NtHSP82* **(K)**, *NtHSF24* **(L)**, *NtHSP90* **(M)**, *NtHSP101* **(N)**, *NtHSP18.2* **(O)**, and *NtHSP17.6* **(P)** responses to heat stress. The data represent the mean ± SE of three biological replicates. ^∗^*P* < 0.05; ^∗∗^*P* < 0.01.

The positive effect of *TaFBA1* on oxidative stress tolerance suggested that *TaFBA1* may be involved in the regulation of ROS homeostasis (Zhou et al., 2015). Therefore, qPCR was used to determine the expression levels of several antioxidative related genes encoding SOD, APX, POD, or CAT in the *TaFBA1*-overexpressing and WT plants (**Figures 8A–D**). Heat stress upregulated the plant transcript levels for all the analyzed genes, but the three OE lines had higher expression levels than the WT plants, particularly for the expression levels of the SOD, POD, and CAT-related genes (**Figures 8A–C**). Furthermore, the POD and CAT expressions were still high after the 3 days recovery period, which suggested that these two genes may play important roles in the recovery process after exposure to heat stress. The trend for *NtAPX* expression was consistent with the APX activity level (**Figures 7C, 8D**).

We then measured the expression levels of eight heat stress marker genes consisting of six HSP genes (*NtHSP101, NtHSP90, NtHSP82, NtHSP70, NtHSP18.2*, and *NtHSP17.6*) and two heat-shock transcription factor (HSF) genes (*NtHSF30* and *NtHSF24*). The expressions of the HSP genes were significantly up-regulated under heat stress as compared with that in the WT plants, but then decreased rapidly after recovery (**Figures 8J,K,M–P**). The expression trend of *NtHSF30* was similar to the six HSP genes (**Figure 8I**). After heat stress, *NtHSF24* expression increased significantly in the transgenic lines, whereas its expression decreased in the WT plants. However, it increased in the WT plants during recovery (**Figure 8L**).

Some genes associated with proline (*NtP5CS*) and stress defense proteins (*NtLEA5, NtERD10C*, and *NtERD10D*) were significantly up-regulated in transgenic plants under heat stress, and then declined after recovery but their expression levels were still higher than that before treatment (**Figures 8E–H**).

### *TaFBA1* Overexpression Increased HSP70 Protein Abundance

**FIGURE 9:**
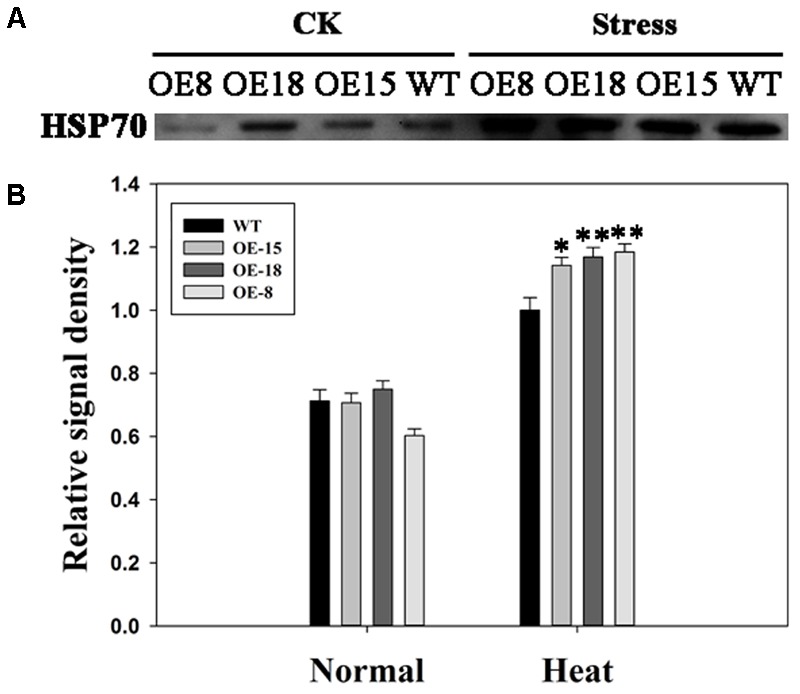
Effects of heat stress on HSP70 protein abundance in the transgenic and WT plants. **(A)** Western blot assay. CK means normal growth condition at 25°C. Stress means plants after 45°C heat stress treatment for 6 h. **(B)** Relative signal density of the HSP70 protein levels. The data represent the mean ± SE of three biological replicates. ^∗^*P* < 0.05; ^∗∗^*P* < 0.01.

From the data in **Figure 8**, we found that the expression level of *NtHSP70* was increased rapidly after heat treatment and the expression level in the transgenic plants was higher than that in WT plants. To explore whether the expression of HSP70 protein had a similar trend as that of the mRNA level of *NtHSP70*, HSP70 protein level in leaves isolated from transgenic and WT plants were studied. Western blot analysis results revealed that HSP70 protein abundance in transgenic plants showed a significant increase as compared with that in WT plants after 6 h heat stress. (**Figures 9A,B**). Thus, *TaFBA1* overexpression increased the HSP70 protein contents in transgenic lines under heat stress.

### TaFBA1 Interacts With an Abiotic Stress Response Protein

**FIGURE 10:**
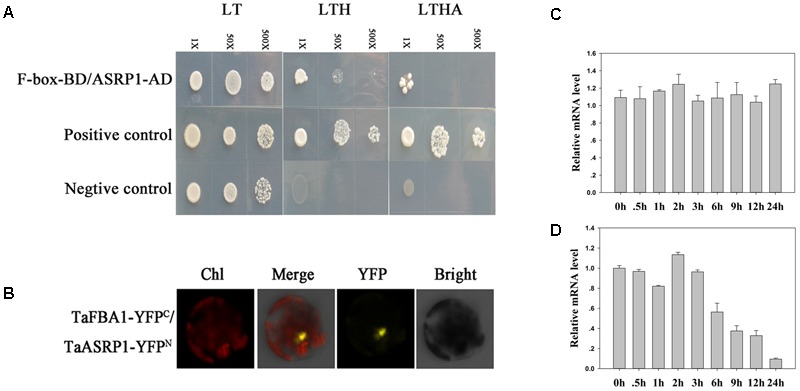
Interaction between TaFBA1 and a deductive stress response protein (named TaASRP1), and the response of *TaASRP1* mRNA expression to heat stress in wheat. **(A)** Y2H analysis of the TaFBA1 and TaASRP1 interaction. The interaction between pGADT7-324 and pGBKT7-332, and pGADT7-322 and pGBKT7-332 proteins were used as a positive control and negative control, respectively. LT, LYH, and LTHA represent the selective media SD-Leu-Trp, SD-Leu-Trp-His, and SD-Leu-Trp-His-Ade, respectively. **(B)** Confirmation of the interaction of TaFBA1 and TaASRP1 by BiFC in *Arabidopsis* protoplasts as shown by a yellow fluorescence signal. nYFP, the construct for the YFP N-terminal fusion expression. cYFP, the construct for the YFP C-terminal fusion expression. Bars = 20 mm. Real time-qPCR analysis of wheat *TaASRP1* mRNA expression responses after 0, 0.5, 1, 2, 3, 6, 9, 12, and 24 h of normal **(C)** and 45°C **(D)** heat treatment. The data represent the mean ± SE of three biological replicates.

Yeast two-hybrid assays were performed to screen for proteins that interact with TaFBA1 in wheat. The full-length cDNA of *TaFBA1* was inserted into the pGBKT7 vector as bait. Positive clones were identified based on their survival on restrictive medium [synthetic dextrose (SD)/-His/-adenine (Ade)/-Trp/-Leu]. A total of 104 positive colonies survived on SD/-His/-Ade/-Trp/-Leu restrictive medium and they were then sequenced. Finally, 15 clones were obtained from *Triticum aestivum* (Supplementary Table S2). In the sequenced colonies, we were particularly interested in a *Triticum aestivum* stress responsive protein 1 (TaASRP1). The cDNA of TaASRP1 was used as prey to further confirm the interaction between TaFBA1 and TaASRP1. An interaction was found between TaFBA1 and TaASRP1 (**Figure 10A**). Moreover, this interaction was further confirmed by BiFC assay (**Figure 10B**).

**FIGURE 11:**
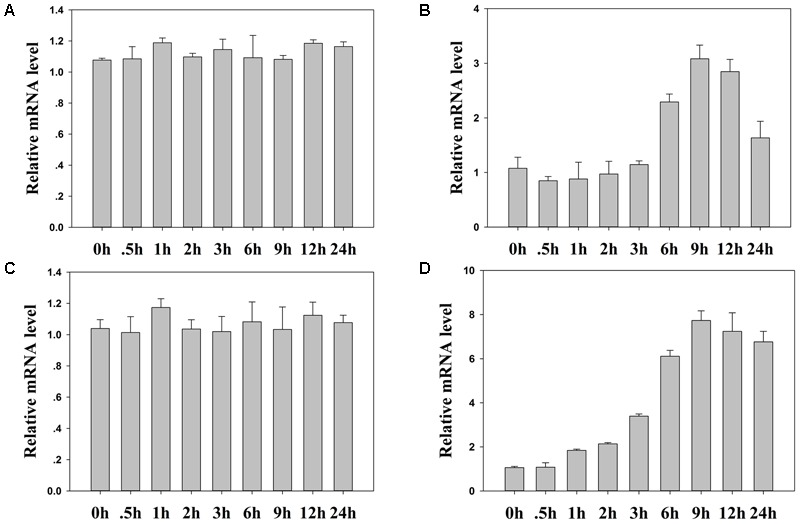
The response of homologous genes in tobacco to heat. **(B)** and **(D)** Real time-qPCR analysis of tobacco *NtFBW2* and *NtASRP1* mRNA expression responses after 0, 0.5, 1, 2, 3, 6, 9, 12, and 24 h of normal **(A)** and 45°C **(C)** heat treatment, respectively. The data represent the mean ± SE of three biological replicates.

We then examined the expression level of *TaASRP1* in response to heat stress in wheat. The results showed that *TaASRP1* expression was induced when the plants were exposed to heat for 2 h, but as the duration of heat stress, its expression was down-regulated while *TaASRP1* expression level in control plants was unchangeable (**Figure 10C**). Furthermore, we found the homologous genes of *TaFBA1* and *TaASRP1* in tobacco, *NtFBW2* and *NtASRP1*. However, different from *TaASRP1* in wheat, the response trend of *NtASRP1* to heat was similar to that of *NtFBW2* in tobacco (**Figure 11**).

## Discussion

### TaFBA1 Is a Subunit of the SCF Complex

F-box proteins are subunits of the SCF complex, which is an E3 ubiquitin ligase. The F-box motif can bind to SKP1 at the N terminus to form a complex that recognizes the target proteins via a protein-protein interaction domain at the C terminus (Sonnberg et al., 2009; Chen et al., 2013). We obtained the F-box *TaFBA1* gene from wheat (*Triticum aestivum* L.) (Zhou et al., 2015) and Y2H assays were undertaken to verify that the protein TaFBA1 is an F-box protein. Our data suggested that TaFBA1, which is a Kelch-type F-box protein, is involved in the formation of an SCF complex with SKP1 and identification of the target protein (**Figure 1**).

### Overexpression of *TaFBA1* Improved the Heat Tolerance of Transgenic Tobacco

Heat is an important environmental stress that affects plant development and production. Under heat stress, some non-functional proteins rapidly accumulate in biological cells. These proteins might influence cellular metabolism and destroy the integrity of the cellular structure (Hellmann and Estelle, 2002). Therefore, it’s important to remove non-functional proteins rapidly if organisms are to adapt to abiotic stresses. The ubiquitin 26S proteasome system (UPS) can degrade non-functional proteins by ubiquitination, in which E3 ligase plays a vital role (Hare et al., 2003; Ding et al., 2015). Various stresses have been reported to induce the expression of E3 ligase genes in plants (Liu et al., 2011; Chen et al., 2013). The F-box may play an important role in the degradation process because it is the key subunit of the E3 ligase SCF complex.

Our previous studies have reported that the stress tolerance of *TaFBA1-*overexpressing tobacco plants had improved (Zhou et al., 2014; Zhao et al., 2017). Consistent with these findings, in the present study, we also observed that heat stress could induce the expression of the *TaFBA1* gene in wheat (**Figure 2**). We found that leaf wilting was more severe in WT plants than in the transgenic lines after exposure to heat stress or following the recovery process in **Figure 3**. Furthermore, the root lengths of the transgenic lines were greater than those in WT plants, and the chlorophyll contents and photosynthetic capacity of the former were greater (**Figure 4**). These results suggest that *TaFBA1* is involved in the improved heat stress tolerance of plants and that its overexpression contributed to this improvement.

### An Active ROS-Scavenging System May Be Involved in the Enhanced Heat Stress Tolerance Shown by Transgenic Tobacco

Various stresses, including drought, high salinity, and extreme temperature, may lead to the over-accumulation of ROS, which can cause damage to plants (Apel and Hirt, 2004; Mittler et al., 2012). Furthermore, membranes are usually the target of heat stress. Under heat stress, membranes are particularly susceptible to ROS-initiated lipid peroxidation reactions (Wang G.D. et al., 2015). The results in **Figure 5** showed that ROS accumulation in the transgenic plants was less than in the WT plants after heat stress. The trypan blue staining assay revealed that the cell damage level was less serious in *TaFBA1*-overexpressing plants than in WT plants. Furthermore, ion leakage and the MDA content of the transgenic plants were significantly lower than in the WT plants (**Figures 5C,D**), which suggested that the improved heat resistance of the *TaFBA1*-overexpressing plants may be due to a stronger ability to scavenge ROS under heat stress conditions. When ROS are produced, plants usually need to take measures to scavenge ROS because their over-accumulation reduces plant growth through enzymatic and non-enzymatic systems (de Azevedo Neto et al., 2006). Therefore, the enhanced activities of the antioxidant enzymes, including SOD, POD, CAT, and APX, might be related to the improved heat tolerance shown by transgenic plants (**Figure 7**). However, APX activity did not increase significantly in this study (**Figure 7C**).

Heat stress can result in intracellular protein oxidation in plant cells. As the main product of protein oxidation, protein carbonyls often irreversibly damage proteins (Kang et al., 2016). The western blot assay showed that less carbonylated proteins were produced in transgenic plants than in WT plants, which suggested that TaFBA1 contributes to alleviating protein oxidation in the transgenic lines (**Figures 6A,B**).

In addition, when the transgenic and WT plants were exposed to drought, salt, and methyl viologen (MV), the activities of antioxidant enzymes were higher in transgenic plants than in WT plants after stress treatment (Zhou et al., 2014, 2015; Kong et al., 2016; Zhao et al., 2017). Based on the results presented in this paper, we speculate that antioxidants may play an important role in the responses of transgenic tobacco exposed to these abiotic stresses. In addition, it implied that this might be a common mechanism of the regulation of tolerance response to stress in plants by *TaFBA1*.

### The Interaction of TaFBA1 With Target Proteins and Related Genes Regulation May Be Involved in the Enhanced Heat Tolerance Seen in Transgenic Plants

The molecular mechanism underlying the plant responses to abiotic stresses might be the regulation of the transcriptional activity of some stress-related genes (Zhu, 2001). The overexpression of *TaFBA1* led to the induction of many stress-related genes, including *NtSOD, NtPOD, NtCAT, NtLEA5, NtP5CS, NtERD10C*, and *NtERD10D*. **Figures 8A–H** show that the expression levels of those genes were higher in the transgenic plants than in WT plants. However, consistent with the APX enzyme activity level, there was no obvious difference in the *NtAPX* expression levels between the transgenic lines and WT plants (**Figure 8D**). This suggested that the up-regulated expression of stress-related genes, with the exception of *NtAPX*, might contribute to the improved stress tolerance in transgenic plants, but the underlying mechanism needs to be explored further.

The complex network of heat-shock responses (HSRs) includes HSPs, HSFs, secondary messengers, ROS, and phytohormone signaling (Kotak et al., 2007; Mittler et al., 2012; Chao et al., 2017). HSPs and ROS-scavenging enzymes are major functional proteins that are induced by heat stress and they are well known as target genes of HS-responsive transcription factors (TFs) (Ohama et al., 2017). HSPs, including HSP100, HSP90, HSP70, HSP60, and small HSPs, function as molecular chaperones and are induced in HSRs (Kotak et al., 2007; Qu et al., 2013). In the present study, we investigated the expression levels of HSR-related genes, including the HSP genes: *NtHSP101, NtHSP90, NtHSP82, NtHSP70, NtHSP18.2*, and *NtHSP17.6*, and the HSF genes: *NtHSF30*, and *NtHSF24* (**Figure 8**). The results indicated that the induction of *NtHSP101, NtHSP90, NtHSP82, NtHSP70, NtHSP18.2, NtHSP17.6, NtHSF30* and *NtHSF24* were more significant in transgenic plants than that in WT plants (**Figures 8I,J,L, M–P**). HSP70 is a widely studied HSP (Hahn et al., 2011). In the present study, the abundance of HSP70 differed significantly between the transgenic lines and the WT plants (**Figure 9**). In addition, these results suggested that *NtHSP70* might play an important role in heat stress tolerance in transgenic tobacco. Moreover, the expression of HSF24 was different between the transgenic and WT plants, indicating the possibility that there is a relationship between TaFBA1 and HSFs. This needs to be studied further.

In the SCF complex, which is a key subunit that targets downstream substrates, the F-box may play a vital role in interactions with proteins. In this study, we carried out a yeast library screening analysis in order to elucidate the possible molecular mechanism underlying the enhancement of tolerance in transgenic plants under heat stress. Among the 15 positive clones acquired from the library screening experiment, we obtained a *Triticum aestivum* stress responsive protein 1 (TaASRP1), and Liu et al. (2015) reported that TaASRP1 response to heat stress. Interested in the function of this protein, we selected it to carry out the next experiments. Both Y2H assay and BiFC assay confirmed the interaction between TaFBA1 and TaASRP1 proteins (**Figures 10A,B**). The qRT-PCR analysis indicated that *TaASRP1* expression was down-regulated after heat treatment (**Figure 10C**). Therefore, we hypothesized that TaFBA1 regulation of TaASRP1 may be important in heat stress tolerance. The enhanced heat tolerance in TaFBA1-overexpressing plants may be caused by the regulation of *TaASRP1* expression. When exposed to continuous heat treatment, the expression level of *TaASRP1* showed a decreasing trend (**Figure 10**). But under heat treatment, the response of *NtASRP1*, homologous gene of *TaASRP1* in tobacco, showed opposite trend to *TaASRP1* (**Figure 10**). Based on this result, we speculated that there might be a relationship between TaFBA1 and TaASRP1 in the response to heat stress. Homologous gene mutants of *TaFBA1* in *Arabidopsis* could be available for carrying out future studies. In the previous study, we got an *Arabidopsis* homologous gene of *TaFBA1* mutant, *atfbw2-4* (Zhao et al., 2017). In addition, we could obtain the information on this relationship by transforming *TaASRP1* to homologous mutants *atfbw2-4*. Moreover, TaFBA1 functions as a member of E3 ubiquitin ligase complex, which was related to the degradation of some proteins. Therefore, further studies on whether TaFBA1 degrades TaASRP1 in wheat, mediated by the UPS, are necessary. As for the different response trend to heat between *TaASRP1* and *NtASRP1*, we speculated that the difference in genetic background between monocot wheat and dicot tobacco might be responsible for the response difference to heat at transcript level.

## Conclusion

The findings of the present study showed that the overexpression of *TaFBA1* improved the heat stress tolerance of transgenic tobacco. The improvement in the ROS-scavenging system through the regulation of stress-responsive gene expression might have contributed to the enhanced stress tolerance shown by the transgenic plants. The expression of HSR related genes was also involved in the regulation of heat stress tolerance in the transgenic lines. The wheat F-box protein, TaFBA1, regulated stress tolerance by interacting with stress-related proteins.

## Author Contributions

QL designed the experiments and performed the experiments. WenqW, WenlW, GZ, YL, and YW provided assistance with the experiments. WeiW provided vital advice on the article. QL wrote the manuscript.

## Conflict of Interest Statement

The authors declare that the research was conducted in the absence of any commercial or financial relationships that could be construed as a potential conflict of interest.
