# Supplementary material for: Wheat F-Box Protein Gene TaFBA1 Is Involved in Plant Tolerance to Heat Stress
Source: Front Plant Sci. 2018 Apr 24;9:521. doi: 10.3389/fpls.2018.00521 (PMC5928245; doi:10.3389/fpls.2018.00521)
Supplement: Supplementary file 1 [file Table_1.DOC]

**Table S1. Primers used in this study**

| Name | Primer sequence (5’-3’) |
| --- | --- |
| ***qRT-PCR*** | |
| TaFBA1-RT-F | AGCAGCAGAACAAGCCTGACCA |
| TaFBA1-RT-R | ACGTGACGTTGGACAGCCTTTG |
| TaASRP1-RT-F | CCAGCATCAAGGACGAGGA |
| TaASRP1-RT-R | GAATACTTACAGGGTGGGACTGG |
| NtActin-RT-F | CATTGGCGCTGAGAGATTCC |
| NtActin-RT-R | GCAGCTTCCATTCCGATCA |
| NtSOD-RT-F | CGGCAATTAGCGGTGACATA |
| NtSOD-RT-R | ATGGCGTCATGTAGCTGTTC |
| NtPOD-RT-F | CTCCATTTCCATGACTGCTTTG |
| NtPOD-RT-R | GTTGGGTGGTGAGGTCTTT |
| NtCAT-RT-F | AGGTACCGCTCATTCACACC |
| NtCAT-RT-R | AAGCAAGCTTTTGACCCAGA |
| NtAPX-RT-F | CAAATGTAAGAGGAAACTCAGAGG |
| NtAPX-RT-R | CAGCCTTGAGCCTCATGGTACCG |
| NtERD10C-RT-F | AACGTGGAGGCTACAGATCG |
| NtERD10C-RT-R | GTTCCTCTTGGGCATGAGTT |
| NtERD10D-RT-F | GAGGACACGGCTGTACCAGT |
| NtERD10D-RT-R | GCGCCACTTCCTCTGTCTT |
| NtP5CS-RT-F | GACACGGACTGATGGAAGATTAG |
| NtP5CS-RT-R | GCACCTGAAGTCACCAGAATAA |
| NtLEA5-RT-F | GTTACCATACCACGTCCCATAG |
| NtLEA5-RT-R | GAGCTAGGACGCTCCATATTT |
| NtHSF30-RT-F | GGTGAGGAATTAGATGAC |
| NtHSF30-RT-R | TATAGGATACTGGAGTTGT |
| NtHSP70-RT-F | CTTAGAAGGTTGAGAACTG |
| NtHSP70-RT-R | GGTAATGGTGGAGTAGAA |
| NtHSP82-RT-F | TTACATTGGATGCTGAACA |
| NtHSP82-RT-R | CTTACACAACAGGCTCAA |
| NtHSF24-RT-F | ATGTCGCAGAGGACAGTTCC |
| NtHSF24-RT-R | GCCGCTCTCGTTCCAAGATA |
| NtHSP90-RT-F | TGAGACTGCCCTCCTCACCT |
| NtHSP90-RT-R | ACCTCCTCCATCTTGCTACCC |
| NtHSP101-RT-F | GGCGATAGATTGCACCAAAGA |
| NtHSP101-RT-R | GCCCCAAGAAAAGGAATGAAC |
| NtHSP18.2-RT-F | TCGAGTGCTCGAATTGATTG |
| NtHSP18.2-RT-R | TAAGGAACTTTCCGCTGCTC |
| NtHSP17.6-RT-F | CTGATTCCGAGCTTCTTTGG |
| NtHSP17.6-RT-R | CCAGGAAGATCCACCTTGAA |
| NtFBW2-RT-F | TGTTGGACGTAAGGGGTTGTT |
| NtFBW2-RT-R | TGTCCCAGCCATTCTTATCGTAG |
| NtASRP1-RT-F | GGCTGATCCCAATTACTCTCTCAC |
| NtASRP1-RT-R | AACCCCTCTTCATCCTTCACTTT |
| ***gene amplification*** | |
| SKP1-F | CACCCAGCGGGCGATGGCGGC |
| SKP1-R | TGCCTAGATGCTCCTCTACT |
| TaFBA1-F | CACCGGAGCAGAGATGGAAGAGCA |
| TaFBA1-R | AGTCGCTGATCTCGCTCCTC |
| Cullin-F | CACCATGACGACGCACGAGCGGAA |
| Cullin-R | TGATCAAGCCAAGTATCGGTA |
| TaASRP-F | CACCATGGACTACTACCGCGAGACC |
| TaASRP-R | GTAGGTTTGGGCATGTCGAT |
