# Supplementary material for: Wheat F-Box Protein Gene TaFBA1 Is Involved in Plant Tolerance to Heat Stress
Source: Front Plant Sci. 2018 Apr 24;9:521. doi: 10.3389/fpls.2018.00521 (PMC5928245; doi:10.3389/fpls.2018.00521)
Supplement: Supplementary file 2 [file Table_2.DOC]

**Table S2. Information about library screening**

| Number | Protein description | GenBank Accessions |
| --- | --- | --- |
| 1 | Shijiazhuang4185 stress responsive protein 1 | JQ923470.1 |
| 2 | WT002_I15 | AK331855.1 |
| 3 | SET6_G07 | AK331140.1 |
| 4 | WT004_K14 | AK332704.1 |
| 5 | tplb0001i01 | AK453337.1 |
| 6 | tplb0008m16 | AK455331.1 |
| 7 | tplb0010i22 | AK455823.1 |
| 8 | tplb0014j16 | AK457008.1 |
| 9 | CK079-F06 | AK446745.1 |
| 10 | CK023-L21 | AK446949.1 |
| 11 | tplb0050a04 | AK451766.1 |
| 12 | tplb0014l13 | AK457042.1 |
| 13 | CK053-A17 | AK446752.1 |
| 14 | tplb0051p16 | AK451937.1 |
| 15 | wle1n.pk0057.g6:fis | BT009213.1 |
